# Fragmentation of care in breast cancer: greater than the sum of its parts

**DOI:** 10.1007/s10549-024-07442-3

**Published:** 2024-08-03

**Authors:** Hadley D. Freeman, Linnea C. Burke, Ja’Neil G. Humphrey, Ashley J. Wilbers, Halley Vora, Rhami Khorfan, Naveenraj L. Solomon, Jukes P. Namm, Liang Ji, Sharon S. Lum

**Affiliations:** 1 Division of Surgical Oncology, Department of Surgery, Loma Linda University, Loma Linda, CA USA; 2 Division of Breast Surgery, Department of Surgery, Washington University, St. Louis, MO USA

**Keywords:** Breast Cancer, Care Fragmentation, Multi-institutional Care, Survival

## Abstract

**Introduction:**

Fragmentation of care (FC, the receipt of care at > 1 institution) has been shown to negatively impact cancer outcomes. Given the multimodal nature of breast cancer treatment, we sought to identify factors associated with FC and its effects on survival of breast cancer patients.

**Methods:**

A retrospective analysis was performed of surgically treated, stage I–III breast cancer patients in the 2004–2020 National Cancer Database, excluding neoadjuvant therapy recipients. Patients were stratified into two groups: FC or non-FC care. Treatment delay was defined as definitive surgery > 60 days after diagnosis. Multivariable logistic regression was performed to identify factors predictive of FC, and survival was compared using Kaplan–Meier and multivariable Cox proportional hazards methods.

**Results:**

Of the 531,644 patients identified, 340,297 (64.0%) received FC. After adjustment, FC (OR 1.27, 95% CI 1.25–1.29) was independently associated with treatment delay. Factors predictive of FC included Hispanic ethnicity (OR 1.04, 95% CI: 1.01–1.07), treatment at comprehensive community cancer programs (OR 1.06, 95% CI: 1.03–1.08) and integrated network cancer programs (OR 1.55, 95% CI: 1.51–1.59), AJCC stage II (OR 1.06, 95% CI 1.05–1.07) and stage III tumors (OR 1.06, 95% CI: 1.02–1.10), and HR + /HER2 + tumors (OR 1.05, 95% CI: 1.02–1.07). Treatment delay was independently associated with increased risk of mortality (HR 1.23, 95% CI 1.20–1.26), whereas FC (HR 0.87, 95% CI 0.86–0.88) showed survival benefit.

**Conclusions:**

While treatment delay negatively impacts survival in breast cancer patients, our findings suggest FC could be a marker for multispecialty care that may mitigate some of these effects.

## Introduction

While there have been vast improvements in diagnostic evaluation and treatment measures resulting in improved outcomes for breast cancer patients over past decades [1, 2], numerous studies have associated treatment delays with disease progression [3] and worse oncologic outcomes in patients with breast cancer [4–8]. As of 2024, the American College of Surgeons Commission on Cancer (CoC) has instituted quality measures to address time to treatment in patients with breast cancer. These recommendations suggest that “in patients with American Joint Committee on Cancer (AJCC) stage I-III breast cancer, the first therapeutic surgery in the non-neoadjuvant setting is performed within and including 60 days of diagnosis” [9].

Fragmentation of care (FC) is defined as receiving care at more than one institution. On a granular level, FC has been shown to negatively impact the broader healthcare system with demonstrable inefficiency as evidenced by higher costs [10, 11], communication gaps [12] longer hospital length of stay [13], higher readmission rates [13, 14], over-utilization of radiological and diagnostic tests [15] and decreased patient satisfaction [16]. These findings may be predicated on various levels of disruption that occur as a result of communication barriers, lack of standardized treatment across multiple sites and deficiencies in care coordination. The effects of FC on timeliness of treatment and survival in cancer patients have been studied and, while a negative connotation exists, results remain largely heterogenous in the literature [10, 11, 17–23]. Retrospective studies have shown FC to negatively impact time to definitive treatment and survival in patients with colon, rectal, liver, and gastric cancers [17–20].

Conversely, FC has been associated with comparable survival in patients with esophageal cancer [21] ovarian cancer [22], and improved survival in those with pancreatic cancer [23], demonstrating benefits may exist for some patients who receive this type of care delivery. While FC remains prevalent in cancer treatment, there is a paucity of data examining breast cancer patients. Therefore, we aimed to identify factors associated with FC, time to treatment, and survival outcomes in patients with breast cancer. We hypothesized that traditionally marginalized patients would have a higher likelihood of receiving FC and that FC is associated with worse outcomes.

## Materials and methods

### Participants

**Fig. 1:**
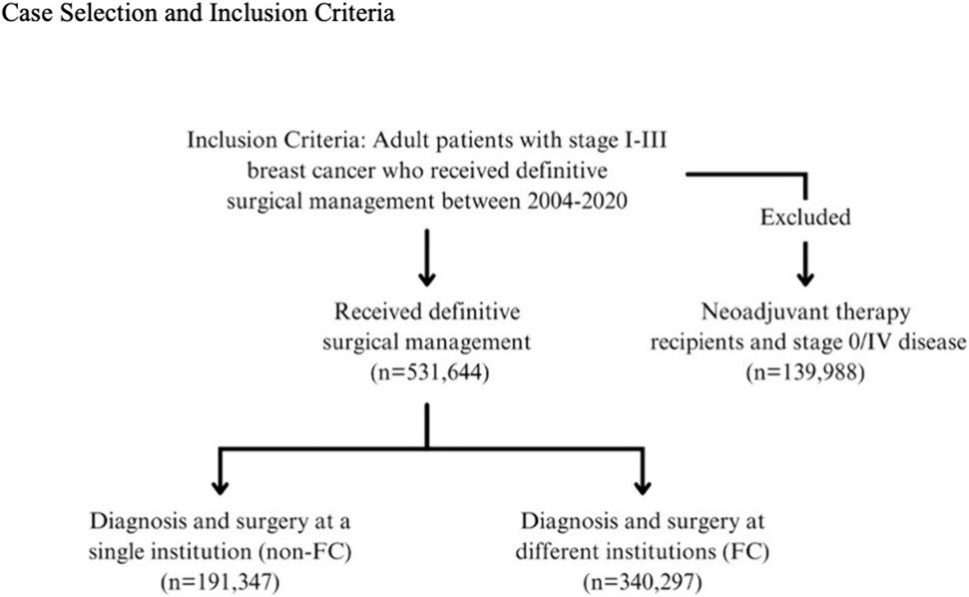
Case selection and inclusion criteria

This study included adult patients with stage I-III invasive breast cancer within the National Cancer Database (NCDB) from 2004 to 2020. The NCDB is a joint project of the Commission on Cancer of the American College of Surgeons and the American Cancer Society and captures 80% of breast cancer cases treated in the United States [24]. Cases were selected that received definitive surgical management. Patients were excluded from this study if they had metastatic disease, received neoadjuvant systemic therapy, had ductal carcinoma in-situ (DCIS), died within 60 days from diagnosis, or received surgery more than 180 days after the initial diagnosis. See Fig. 1 for complete case selection criteria.

### Data collection

Patient demographics including age, race and ethnicity, insurance status, treating facility type, income, education level, AJCC stage, receptor status, and Charlson-Deyo score were collected. The presence or absence of FC was defined by the class of case variable [25]. Patients who had their diagnosis and all treatment at the same facility were considered non-FC (class of case 12, 14) and those diagnosed and treated at different facilities were considered FC (class of case 11, 13, 21, 22). Time from diagnosis to definitive surgery was categorized as treatment delay (> 60 days) and no delay (≤ 60 days). Overall survival was determined by date of diagnosis to date of death or last follow-up.

### Data analysis

Patients were stratified into two cohorts for analysis of FC vs non-FC and treatment delay vs. no delay and compared by Chi-squared tests. Multivariable logistic regression was performed to identify factors associated with FC and delay in treatment. Cox proportional hazards models were used to estimate mortality hazards ratios (HR) with 95% confidence interval (CI) limits. Kaplan–Meier survival curves with log-rank tests were used to compare survival times. A sensitivity analysis of propensity score distributions for FC vs non-FC cohorts yielded similar probabilities for these large sample sizes; therefore, propensity score matching was not performed. All tests were two-sided, performed using a significance level of *α* = 0.05. Data analyses were conducted using SAS 9.4 (SAS Institute Inc. 2023. Base SAS® 15.3 Utilities. Cary, NC: SAS Institute Inc.). Due to the de-identified nature of NCDB data, this study was deemed exempt from formal review by the Loma Linda University Institutional Review Board.

## Results

### Baseline patient, tumor and treating facility characteristics

A total of 531,644 patients met inclusion criteria. Overall, 340,297 (64.0%) received FC while 191,347 (36.0%) received non-FC care. Baseline patient demographics, tumor characteristics and treating facility types are shown in Table 1. Patients receiving FC were more likely to be younger (mean age 63.2 vs 65.4 years, *p* < 0.0001), non-Hispanic White (82.5% vs 81.2%, *p* < 0.001), and have private insurance/managed care (49.9% vs 43.6% (*p* < 0.0001). Receipt of FC was more common in patients with AJCC stage II or stage III disease (26.5% vs 25.7%, *p* < 0.0001) and hormone receptor (HR) positive tumors (88.0% vs 87.1%, *p* < 0.0001). Treatment delay was more prevalent in patients with FC (12.1% vs 9.9%, *p* < 0.0001).

**Table 1:** Baseline patient demographics, tumor characteristics, treating facility types of patients with stage I–III invasive breast cancer receiving definitive surgical treatment

Variable	Fragmented care *N* = 340,297 (%)	Non-fragmented care *N* = 191,347 (%)	*p*-value
Race and ethnicity			
Asian/other	14,355 (4.2%)	7264 (3.8%)	< 0.0001
Non-hispanic black	28,678 (8.4%)	20,369 (10.7%)	
Hispanic	16,588 (4.9%)	8329 (4.4%)	
Non-hispanic white	280,676 (82.5%)	155,385 (81.2%)	
Insurance			
Not insured	3624 (1.1%)	2945 (1.5%)	< 0.0001
Private insurance/managed care	169,773 (49.9%)	83,532 (43.6%)	
Medicaid	16,489 (4.9%)	10,012 (5.2%)	
Medicare	146,764 (43.1%)	93,420 (48.8%)	
Other government	3647 (1.1%)	1438 (0.8%)	
Facility type			
Community cancer program	24,330 (7.2%)	15,673 (8.2%)	< 0.0001
Comprehensive community cancer program	144,328 (42.4%)	86,019 (45.0%)	
Academic/research program	95,464 (28.1%)	58,604 (30.6%)	
Integrated network cancer program	76,175 (22.4%)	31,051 (16.2%)	
Income			
< $46,277	43,185 (12.7%)	28,915 (15.1%)	< 0.0001
$46,277–$57,856	66,837 (19.6%)	39,342 (20.6%)	
$57,857–$74,062	80,329 (23.6%)	46,932 (24.5%)	
$74,063 +	149,946 (44.1%)	76,158 (39.8%)	
Education			
< 5%	91,781 (26.7%)	50,578 (26.2%)	< 0.0001
5.0%–9.0%	105,782 (30.8%)	57,801 (29.9%)	
9.1%–15.2%	88,084 (25.7%)	52,187 (27.0%)	
> / = 15.3%	57,644 (16.8%)	32,771 (17.0%)	
AJCC stage			
I	250,036 (73.5%)	142,132 (74.3%)	< 0.0001
II	83,143 (24.4%)	45,127 (23.6%)	
III	7118 (2.1%)	4088 (2.1%)	
Receptor status			
HR + HER2 +	17,119 (5.0%)	9147 (4.8%)	< 0.0001
HR + HER2-	282,440 (83.0%)	157,449 (82.3%)	
HR-HER2 +	8944 (2.6%)	5257 (2.8%)	
Triple Negative	31,794 (9.3%)	19,494 (10.2%)	
Charlson-Deyo score			
0	280,029 (82.3%)	151,432 (79.1%)	< 0.0001
1	47,019 (13.8%)	29,744 (15.5%)	
2	9627 (2.8%)	7042 (3.7%)	
3	3622 (1.1%)	3129 (1.6%)	
Time to treatment			
< / = 60d	299,009 (87.9%)	172,320 (90.1%)	< 0.0001
> 60d	41,288 (12.1%)	19,027 (9.9%)	

Patients diagnosed and treated at a single institution were more likely to receive care at a comprehensive community cancer program (45.0% vs 42.4%, *p* < 0.0001) or academic/research program (30.6% vs 28.1%, *p* < 0.0001), have stage I disease (74.3% vs 73.5%, *p* < 0.0001), and have surgery within 60 days of diagnosis (90.1% vs 87.9%, *p* < 0.0001).

### Factors associated with treatment delay

After adjustment, multiple factors were independently associated with treatment delay. Patient characteristics included Hispanic (OR 1.82, 95% CI: 1.76–1.88), non-Hispanic Black (OR 1.82, 95% CI: 1.77–1.87), and Asian/other race and ethnicities (OR 1.25, 95% CI: 1.20–1.31) as well as those with Medicaid insurance (OR 1.89, 95% CI: 1.83–1.95), uninsured status (OR 1.72, 95% CI: 1.62–1.83), Medicare (OR 1.11, 95% CI: 1.08–1.14), and other Government insurance (or 1.30, 95% CI: 1.20–1.41), and lower education levels, with the greatest risk in patients residing in zip codes with at or above 15.3% without high school diplomas (OR 1.47, 95% CI: 1.43–1.52). In terms of treatment facility, those treated at academic/research programs (OR 1.59, 95% CI: 1.53–1.65), integrated network cancer programs (OR 1.42, 95% CI: 1.36–1.48), and comprehensive community cancer programs (OR 1.17, 95% CI: 1.13–1.22) had increased odds of treatment delay compared to community cancer programs. Patients with AJCC stage II (OR 1.15, 95% CI: 1.13–1.18) and AJCC stage III tumors (OR 1.15, 95% CI: 1.09–1.22) had increased odds of treatment delay compared to AJCC stage I tumors. Receipt of FC (OR 1.27, 95% CI: 1.25–1.29) was independently associated with delayed treatment as well (Table 2).

**Table 2:** Adjusted multivariable analysis of factors associated with treatment delay in patients with stage I–III invasive breast cancer receiving definitive surgical treatment

Variable	OR	95% CI	*p*-value
Age (yr)	0.99	0.99–0.99	< 0.0001
Race and ethnicity			
Asian/other	1.25	1.20–1.31	< 0.0001
Non-hispanic black	1.82	1.77–1.87	< 0.0001
Hispanic	1.82	1.76–1.88	< 0.0001
Non-hispanic white	Ref		
Insurance			
Not insured	1.72	1.62–1.83	< 0.0001
Medicaid	1.88	1.82–1.94	< 0.0001
Medicare	1.11	1.08–1.14	< 0.0001
Other government	1.30	1.20–1.41	< 0.0001
Private insurance/managed care	Ref		
Facility type			
Community cancer program	Ref		
Comprehensive community cancer program	1.17	1.13–1.22	< 0.0001
Academic/research program	1.80	1.73–1.87	< 0.0001
Integrated network cancer program	1.42	1.36–1.48	< 0.0001
Income			
< $46,277	0.76	0.77–0.82	< 0.0001
$46,277–$57,856	0.87	0.85–0.89	< 0.0001
$57,857–$74,062	0.95	0.92–0.97	< 0.0001
$74,063 +	Ref		
Education			
< 5%	Ref		
5.0%–9.0%	1.15	1.12–1.18	< 0.0001
9.1%–15.2%	1.28	1.25–1.32	< 0.0001
>/= 15.3%	1.47	1.43–1.52	< 0.0001
AJCC stage			
I	Ref		
II	1.15	1.13–1.18	< 0.0001
III	1.15	1.09–1.22	< 0.0001
Receptor status			
HR + HER2 +	1.00	0.96–1.04	0.8086
HR + HER2-	Ref		
HR-HER2 +	0.88	0.83–0.92	< 0.0001
Triple negative	0.74	0.72–0.76	< 0.0001
Charlson-Deyo score			
0	Ref		
1	1.06	1.04–1.09	< 0.0001
2	1.24	1.18–1.30	< 0.0001
3	1.48	1.38–1.58	< 0.0001
Fragmented care			
Yes	1.27	1.25–1.29	< 0.0001
No	Ref		

Younger age (OR 0.99, 95% CI: 0.99–0.99), lower income status– with levels of less than $46,277 having lowest odds (OR 0.81, 95% CI 0.78–0.83), and tumors with HR-/HER2 + receptor status (OR 0.87, 95% CI: 0.82–0.91) and triple negative receptor status (OR 0.73, 95% CI: 0.71–0.76) were independently associated with lower likelihood of treatment delay (Table 2).

### Factors associated with fragmentation of care

After adjustment, multiple factors were independently associated with FC. Patient characteristics included Hispanic ethnicity (OR 1.04, 95% CI: 1.01–1.07), those with Medicare insurance (OR 1.04, 95% CI: 1.02–1.06), other Government insurance (OR 1.35, 95% CI: 1.27–1.43), and lower education levels, with the greatest risk in patients residing in zip codes with at or above 15.3% without high school diplomas (OR 1.28, 95% CI 1.26–1.31). Compared to treatment at community cancer programs, treatment at integrated network cancer programs (OR 1.55, 95% CI: 1.51–1.59) and comprehensive community cancer programs (OR 1.06, 95% CI: 1.03–1.08) were independently associated with FC. Patients with AJCC stage II (OR 1.06, 95% CI: 1.05–1.07) and stage III (OR 1.06, 95% CI: 1.02–1.10) tumors and HR + /HER2 + receptor status (OR 1.05, 95% CI: 1.02–1.07) also had increased odds of receiving FC (Table 3).

**Table 3:** Adjusted multivariable analysis of factors associated with fragmentation of care in patients with stage I–III invasive breast cancer receiving definitive surgical treatment

Variable	Odds ratio	95% CI	*p*-value
Age (yr)	0.98	0.98–0.98	< 0.0001
Race and ethnicity			
Asian/other	1.00	0.97–1.03	0.7362
Non-hispanic black	0.78	0.77–0.80	< 0.0001
Hispanic	1.04	1.01–1.07	0.0161
Non-hispanic white	Ref		
Insurance			
Medicaid	0.83	0.80–0.85	< 0.0001
Medicare	1.04	1.02–1.06	< 0.0001
Not insured	0.62	0.58–0.65	< 0.0001
Other government	1.35	1.27–1.43	< 0.0001
Private insurance/managed care	Ref		
Facility type			
Academic/research program	0.99	0.96–1.01	0.2467
Community cancer program	Ref		
Comprehensive community cancer program	1.06	1.03–1.08	< 0.0001
Integrated network cancer program	1.55	1.51–1.59	< 0.0001
Income			
< $46,277	0.73	0.72–0.75	< 0.0001
$46,277–$57,856	0.84	0.82–0.85	< 0.0001
$57,857–$74,062	0.84	0.83–0.86	< 0.0001
$74,063 +	Ref		
Education			
< 5%	Ref		
5.0–9.0%	1.11	1.09–1.13	< 0.0001
9.1%-15.2%	1.13	1.11–1.15	< 0.0001
> / = 15.3%	1.28	1.26–1.31	< 0.0001
AJCC stage			
I	Ref		
II	1.06	1.05–1.07	< 0.0001
III	1.06	1.02–1.10	0.0040
Receptor status			
HR + HER2 +	1.05	1.02–1.07	0.0010
HR + HER2-	Ref		
HR-HER2 +	0.95	0.92–0.98	0.0039
Triple negative	0.94	0.92–0.95	< 0.0001
Charlson-Deyo score			
0	Ref		
1	0.92	0.90–0.93	< 0.0001
2	0.82	0.79–0.85	< 0.0001
3	0.71	0.68–0.75	< 0.0001
Time to treatment			
< / = 60d	0.79	0.78–0.81	< 0.0001
> 60d	Ref		

Patient factors predictive of non-FC care included younger age (OR 0.98, 95% CI 0.98–0.98), non-Hispanic Black race and ethnicity (OR 0.78, 95% CI: 0.77–0.80), uninsured status (OR 0.62, 95% CI: 0.58–0.65) those with Medicaid insurance (OR 0.83, 95% CI: 0.80–0.85), and those with lower income status—with income lower than $46,277 having greatest association (OR 0.73, 95% CI: 0.72–0.75). Tumor characteristics including HR-/HER2 + receptor status (OR 0.95, 95% CI: 0.92–0.98) and triple negative receptor status (OR 0.94, 95% CI: 0.92–0.95) had lower odds of FC. Patients receiving definitive surgical treatment within 60 days of diagnosis also had lower likelihood of FC (Table 3).

### Survival

**Fig. 2:**
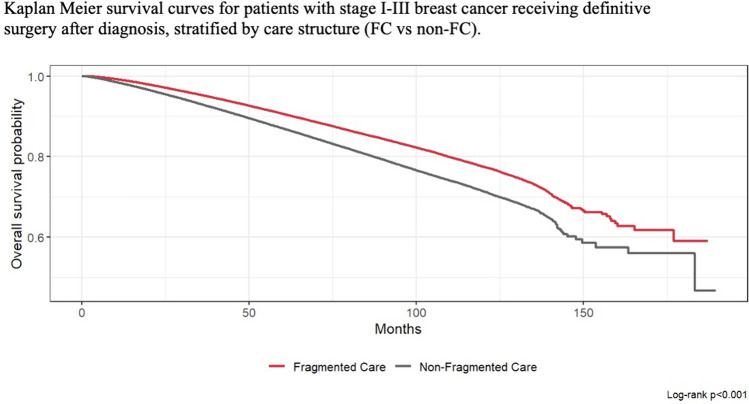
Kaplan Meier survival curves for patients with stage I-III breast cancer receiving definitive surgery after diagnosis, stratified by care structure (FC vs non-FC)

Patient factors independently associated with lower likelihood of mortality included Hispanic (HR 0.73, 95% CI: 0.70–0.76) and Asian/ other race and ethnicities (HR 0.71, 95% CI: 0.68–0.75). Compared to treatment at community cancer programs, treatment at academic/research programs (HR 0.80, 95% CI: 0.78–0.82), comprehensive community cancer programs (HR 0.93, 95% CI: 0.91–0.95), and integrated network cancer programs (HR 0.90, 95% CI: 0.88–0.93) were associated with reduced mortality (Table 4). Patients receiving FC had lower likelihood of mortality compared to those with non-FC care (HR 0.87, 95% CI: 0.86–0.88) as shown in Fig. 2.

**Table 4:** Adjusted multivariable proportional hazards model of factors associated with mortality in patients with stage I-III invasive breast cancer receiving definitive surgical treatment

Variable	HR	95% CI	*p*-value
Age	1.07	1.07–1.07	< 0.0001
Race and ethnicity			
Asian/other	0.71	0.68–0.75	< 0.0001
Non-hispanic black	1.08	1.05–1.10	< 0.0001
Hispanic	0.73	0.70–0.76	< 0.0001
Non-hispanic white	Ref		
Insurance			
Not insured	1.50	1.40–1.61	< 0.0001
Medicaid	1.75	1.69–1.82	< 0.0001
Medicare	1.18	1.16–1.21	< 0.0001
Other government	1.19	1.10–1.29	< 0.0001
Private insurance/managed care	Ref		
Facility type			
Comprehensive community cancer program	0.93	0.91–0.95	< 0.0001
Academic/research program	0.80	0.78–0.82	< 0.0001
Integrated network cancer program	0.90	0.88–0.93	< 0.0001
Community cancer program	Ref		
Income			
< $46,277	1.27	1.24–1.30	< 0.0001
$46,277-$57,856	1.20	1.18–1.23	< 0.0001
$57,857-$74,062	1.11	1.09–1.13	< 0.0001
$74,063 +	Ref		
Education			
< 5%	Ref		
5.0%-9.0%	1.09	1.06–1.11	< 0.0001
9.1%-15.2%	1.10	1.08–1.13	< 0.0001
> / = 15.3%	1.07	1.04–1.10	< 0.0001
AJCC stage			
I	Ref		
II	1.80	1.77–1.82	< 0.0001
III	3.46	3.36–3.56	< 0.0001
Receptor status			
HR + HER2 +	1.21	1.18–1.24	< 0.0001
HR + HER2-	Ref		
HR-HER2 +	1.24	1.20–1.29	< 0.0001
Triple negative	1.62	1.59–1.66	< 0.0001
Charlson-Deyo score			
0	Ref		
1	1.44	1.41–1.46	< 0.0001
2	2.09	2.03–2.15	< 0.0001
3	3.01	2.90–3.13	< 0.0001
Time to treatment			
< / = 60d	Ref		
> 60d	1.23	1.20–1.26	< 0.0001
Fragmented care			
Yes	0.87	0.86–0.88	< 0.0001
No	Ref

**Fig. 3:**
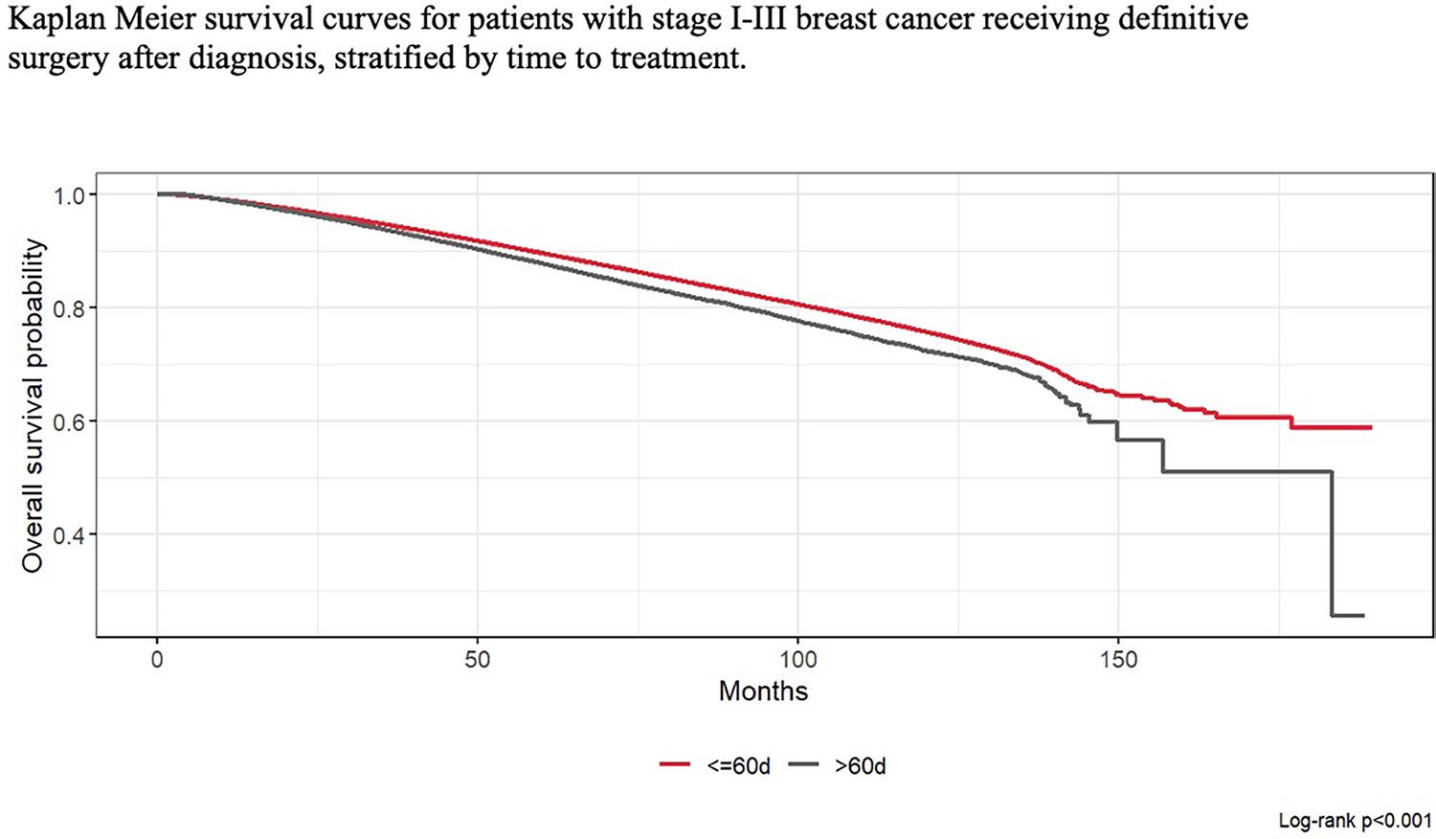
Kaplan Meier survival curves for patients with stage I-III breast cancer receiving definitive surgery after diagnosis, stratified by time to treatment (<=60 vs > 60 days)

Delay in treatment greater than 60 days was independently associated with increased risk of mortality (HR 1.23, 95% CI 1.20–1.26) as shown in Fig. 3. Patient age (HR 1.07, 95% CI: 1.07–1.07), non-Hispanic Black race and ethnicity (HR 1.08, 95% CI: 1.05–1.10), Medicaid insurance (HR 1.75, 95% CI: 1.69–1.81), lower income—with the greatest risk at income level below $46,277 (HR 1.27, 95% CI: 1.24–1.30), lower education levels—with the greatest risk in patients residing in zip codes with 9.1%–15.2% without high school diplomas (OR 1.10, 95% CI: 1.08–1.13), AJCC stage II tumors (HR 1.80, 95% CI: 1.77–1.82), AJCC stage III tumors (HR 3.46, 95% CI: 3.36–3.56), HR+/HER2 + receptor status (HR 1.21, 95% CI: 1.18–1.24), and Charlson-Deyo score I (HR 1.44, 95% CI: 1.41–1.46), Charlson-Deyo score II (HR 2.09, 95% CI: 2.03–2.15) and Charlson-Deyo score III (HR 3.01, 95% CI: 2.90–3.13) were independently associated with higher risk of mortality (Table 4).

## Discussion

To our knowledge, this is the first observational study utilizing NCDB data to evaluate care fragmentation and its implications on timeliness of treatment and survival in patients with stage I-III breast cancer in the United States. This study highlights the various patient characteristics, treating facility subtypes, and tumor properties associated with both care fragmentation and treatment delay. Furthermore, we explore the independent effects on survival according to treatment delay and FC. In this large cohort of breast cancer patients receiving upfront definitive surgery after diagnosis between 2004 and 2020, the majority received FC. While care fragmentation was associated with treatment delay and multiple patient, treating facility, and tumor factors exhibited overlap between FC and delayed treatment, FC was unexpectedly found to be independently associated with survival benefit. Conversely, treatment delay demonstrated increased risk of mortality. Despite the conceptual negative connotation surrounding fragmentation of care in patients with cancer, our findings suggest FC is a highly complex topic with interwoven benefits to both patients and the overall healthcare system and deserves further investigation.

Upon review of the existing literature, our study population demonstrated a higher proportion (63.9%) of breast cancer patients receiving FC compared to those with esophageal cancer (57.6%) [21], thyroid cancer (53.3%) [8], gastric cancer (48.7%) [18], ovarian cancer (36.8%) [22], rectal cancer (35%) [20], hepatocellular carcinoma (27.4%) [19] and pancreatic cancer (24.1%) [23]. Given the varying degrees of diagnostic, medical, and surgical complexity in treating patients with cancer, it is reasonable to conclude that the rates of FC across the cancer spectrum will differ. For example, the CoC developed the National Accreditation Program for Rectal Cancer (NAPRC) to standardize the treatment of rectal cancer and improve outcomes [26]. Once patients are seen by a treating physician at one of these accredited programs, it is possible they will continue their care at the NAPRC institution, lowering the rates of FC. Furthermore, volume-outcomes relationships have been described in patients with complex gastrointestinal and hepatobiliary malignancies [27–29] requiring a high degree of surgical expertise. Receipt of care at a centralized institution may be more likely in this cohort of patients unlike those with breast cancer, who are treated by a vast array of general and sub-specialty surgeons nationwide.

The majority of breast cancer patients receiving FC were non-Hispanic White (82.5%) and the highest proportion had private insurance/managed care (49.8%), which parallels other published reports [20, 21]. Our study found that, among patients with breast cancer, comprehensive community cancer programs had the highest proportion of FC (42.4%) followed by academic/research programs (28.1%), whereas a retrospective analysis on patients with rectal cancer reported the highest proportion of FC to be within academic institutions (38%) [20].

According to our multivariate analysis, Hispanic ethnicity was shown to be independently associated with receipt of FC which, to our knowledge, has not been demonstrated in previous studies. This study corroborates a retrospective NCDB analysis on effects of care fragmentation in patients with pancreatic cancer [23], which found non-Hispanic Black race and ethnicity and lack of private insurance to be negatively predictive of FC. Our study found lower odds of FC with lower income levels, substantiated by existing data in which higher income levels were independently associated with receipt of FC [22, 23].

Regarding tumor biology, this study found AJCC stage II and AJCC stage III disease to be independently associated with FC. A paucity of comparative data on this subset of breast cancer research exists. According to a prospective study by Doose et al. [30] which analyzed care fragmentation on Black breast cancer patients with comorbidities in New Jersey, cancer stage was not independently associated with FC. It is plausible to infer that patients with more advanced staged tumors are more likely to receive FC due to the need for a specialized, multidisciplinary approach after diagnosis. Additionally, type of insurance coverage, or lack thereof, can be a barrier to external referrals which could lower the likelihood of FC in certain patients. In contrast, patients with private insurance may be choice enabled to seek care at a variety of institutions.

In terms of structural aspects of care, breast cancer patients undergoing care at comprehensive community cancer programs and integrated network cancer programs had higher likelihood of receiving FC, whereas academic/research programs were independently associated with lower odds of FC. Data on this topic are mixed. While some studies have found academic institutions and high-volume centers to be independently associated with coordinated care (receipt of care at one institution) [21], others have shown treatment at non-academic centers to be negatively predictive of FC [23]. Geographical and population characteristics may contribute to this variability. While specific geographical data were not included in this study, previous reports have shown that travel distance is independently associated with FC [18, 31]. Some states which are more densely populated may have medical systems that comprise a higher proportion of CoC academic institutions, thus lowering the rate of FC in those regions. Referral bias may underline these findings as well. Cancer patients diagnosed within systems that have well-established, streamlined in-hospital referral patterns to oncology specialists may be less likely to receive FC compared to their counterparts.

Despite studies investigating the relationship between care fragmentation, timeliness of treatment, and survival in other malignancies, these results are highly variable and likely dependent upon multiple factors specific to each disease process and treatment profile. While our study showed FC to be independently associated with both treatment delay and improved survival, treatment delay in itself was associated with increased risk of mortality. This finding aligns with previous studies which have established the negative impact on survival in patients with breast cancer experiencing delayed treatment [4–7, 32]. Similar to our results on FC, time to treatment, and survival, a retrospective analysis by Rhodin et al. [21] found FC to be associated with delay in surgical intervention after neoadjuvant chemotherapy in esophageal cancer patients; however, survival was preserved [21]. Brown et al. [33] described longer time to initiation of adjuvant therapy after surgery in patients with pancreatic cancer experiencing FC, although no difference in overall survival was observed [33]. In patients with stage I-III differentiated thyroid cancer, FC was associated with minor treatment delays and improved 5-year overall survival [8]. Conversely, FC has been associated with increased mortality in patients with gastric cancer [18], rectal cancer [20], hepatocellular carcinoma [19], and breast cancer patients in Colombia [11]. These findings suggest the relationships between FC, time to treatment, and survival have intricacies which are not linear. Patients harboring more aggressive disease may not be well suited for the expected time delays experienced with referring to other centers, thus having the entirety of their treatment at a single facility and contributing to our findings of increased mortality. For patients who are referred to another institution, those with more aggressive tumor biology may experience more of the detrimental effects related to treatment delays and care fragmentation compared to those with more indolent tumors. The independent effects of FC and treatment delays on survival as related to neoadjuvant therapy, definitive surgery, and adjuvant therapies are presumably multifactorial and highly specific to each patient and malignancy type. Lastly, compared to the work by Gamboa et al. [11] which described increased mortality experienced by breast cancer patients receiving FC in Colombia, our study designs and patient populations are quite dissimilar which could explain variability in the results of these two studies.

Several limitations which are inherent to the retrospective nature of this study exist. Given the retrospective design, there are likely unmeasured confounding factors relating to our observations on FC. While the NCDB is a robust database, missing information and potential errors exist. However, the NCDB contains 80% of incident breast cancer cases in the United States and the Commission on Cancer performs regular audits of reporting facilities for data quality assurance” [24, 34]. Specific to the breast cancer population, fine details pertaining to clinical data including patient risk factors, presenting symptoms, methods of diagnosis, referral patterns, and follow-up care structure are not captured in the NCDB. Geographical data (i.e., travel distance, residential grouping) was not included in this study yet may be a confounding factor as this has been associated with FC, as mentioned in the discussion. Furthermore, we excluded all neoadjuvant therapy recipients: whether our findings are applicable to patients requiring neoadjuvant systemic treatment cannot be determined. Our study utilized NCDB data ranging from years 2004–2020. Multimodal treatments and the delivery of health care services have changed dramatically over this period and temporal changes were not addressed. This time period also included the first year of the COVID-19 pandemic, during which NCDB data were disrupted [35]. Therefore, results from year 2020 must be carefully interpreted and it is possible breast cancer cases were underreported during this time. While observed small percentage point differentials between cohorts were statistically significant, these findings may not be clinically significant. Access to NCDB data lags real time reporting of cases by tumor registrars, as updates to cases diagnosed in the prior months can be submitted later and participant user files are released in subsequent years [36]. Finally, as NCDB data are derived from CoC accredited institutions, breast cancer patients treated in facilities not accredited by the CoC lack visibility in this study. Patients at the extremes of age, those of Hispanic, Black, Asian or Pacific Islander, American Indian or Alaskan Native race and ethnicity are therefore underrepresented in the NCDB.

## Conclusion

As centralization of cancer care has been a focus in recent years, FC-related outcomes have emerged as an area of interest. This large, retrospective NCDB analysis on fragmentation of care in patients with stage I-III breast cancer contributes to the existing literature and further characterizes the impact of FC on this subgroup of patients. Although FC was associated with treatment delay, mortality benefit was also observed. While there are well recognized disparities associated with FC in many cancer types, within the breast cancer patient population, the distinction should be made between what FC is, and what it is not. FC is not necessarily the absence of quality care; rather, it likely encompasses a number of benefits related to multimodal and multispecialty approaches to cancer care and guideline-concordant treatment. Further investigation is warranted to delineate these effects, particularly by facility type. In conclusion, we endorse reframing the phrase “fragmentation of care” to a more unbiased term such as “multi-institutional care”, as denoted in some studies [21], to encompass the broader scope of this type of care delivery.

## Data Availability

No datasets were generated or analysed during the current study.

## References

[1] Toriola AT, Colditz GA (2013) Trends in breast cancer incidence and mortality in the United States: implications for prevention. Breast Cancer Res Treat 138(3):665–673. 10.1007/s10549-013-2500-7 23546552 10.1007/s10549-013-2500-7

[2] Chu KC, Tarone RE, Kessler LG et al (1996) Recent trends in U.S. breast cancer incidence, survival, and mortality rates. J Natl Cancer Inst 88(21):1571–1579. 10.1093/jnci/88.21.1571 8901855 10.1093/jnci/88.21.1571

[3] Hills N, Leslie M, Davis R et al (2021) Prolonged time from diagnosis to breast-conserving surgery is associated with upstaging in hormone receptor-positive invasive ductal breast carcinoma. Ann Surg Oncol 28(11):5895–5905. 10.1245/s10434-021-09747-9 33748899 10.1245/s10434-021-09747-9 PMC7982278

[4] Richards MA, Westcombe AM, Love SB, Littlejohns P, Ramirez AJ (1999) Influence of delay on survival in patients with breast cancer: a systematic review. Lancet 353(9159):1119–1126. 10.1016/s0140-6736(99)02143-1 10209974 10.1016/s0140-6736(99)02143-1

[5] Eaglehouse YL, Georg MW, Shriver CD, Zhu K (2019) Time-to-surgery and overall survival after breast cancer diagnosis in a universal health system. Breast Cancer Res Treat 178(2):441–450. 10.1007/s10549-019-05404-8 31414244 10.1007/s10549-019-05404-8

[6] Hanna TP, King WD, Thibodeau S et al (2020) Mortality due to cancer treatment delay: systematic review and meta-analysis. BMJ 371:4087. 10.1136/bmj.m4087 10.1136/bmj.m4087 PMC7610021 33148535

[7] Pathak R, Leslie M, Dondapati P et al (2023) Increased breast cancer mortality due to treatment delay and needle biopsy type: a retrospective analysis of SEER-medicare. Breast Cancer 30(4):627–636. 10.1007/s12282-023-01456-3 37130988 10.1007/s12282-023-01456-3 PMC10284985

[8] Greenberg JA, Thiesmeyer JW, Egan CE et al (2022) Care fragmentation in patients with differentiated thyroid cancer. World J Surg 46(12):3007–3016. 10.1007/s00268-022-06712-9 36038731 10.1007/s00268-022-06712-9

[9] Quality of Care Measures. ACS. https://www.facs.org/quality-programs/cancer-programs/national-cancer-database/quality-of-care-measures/#:~:text=For%20patients%20with%20AJCC%20Clinical Accessed April 3 2024

[10] Skolarus TA, Zhang Y, Hollenbeck BK (2012) Understanding fragmentation of prostate cancer survivorship care: implications for cost and quality. Cancer 118(11):2837–2845. 10.1002/cncr.26601 22370955 10.1002/cncr.26601 PMC4860006

[11] Gamboa Ó, Buitrago G, Patiño AF et al (2023) Fragmentation of care and its association with survival and costs for patients with breast cancer in Colombia. JCO Glob Oncol 9:e2200393. 10.1200/GO.22.00393 37167575 10.1200/GO.22.00393 PMC10497266

[12] Leggett N, Emery K, Rollinson TC et al (2023) Fragmentation of care between intensive and primary care settings and opportunities for improvement. Thorax 78(12):1181–1187. 10.1136/thorax-2023-220387 37620046 10.1136/thorax-2023-220387

[13] Snow K, Galaviz K, Turbow S (2020) Patient outcomes following interhospital care fragmentation: a systematic review. J Gen Intern Med 35(5):1550–1558. 10.1007/s11606-019-05366-z 31625038 10.1007/s11606-019-05366-z PMC7210367

[14] Missios S, Bekelis K (2016) Outpatient continuity of care and 30-day readmission after spine surgery. Spine J 16(11):1309–1314. 10.1016/j.spinee.2016.06.012 27349630 10.1016/j.spinee.2016.06.012

[15] Kern LM, Seirup JK, Casalino LP, Safford MM (2017) Healthcare fragmentation and the frequency of radiology and other diagnostic tests: a cross-sectional study. J Gen Intern Med 32(2):175–181. 10.1007/s11606-016-3883-z 27796694 10.1007/s11606-016-3883-z PMC5264678

[16] Fan VS, Burman M, McDonell MB, Fihn SD (2005) Continuity of care and other determinants of patient satisfaction with primary care. J Gen Intern Med 20(3):226–233. 10.1111/j.1525-1497.2005.40135.x 15836525 10.1111/j.1525-1497.2005.40135.x PMC1490082

[17] Choi DW, Kim S, Kim DW, Han KT (2022) Fragmentation of care and colorectal cancer survival in South Korea: comparisons according to treatment at multiple hospitals. J Cancer Res Clin Oncol 148(9):2323–2333. 10.1007/s00432-022-04035-9 35522291 10.1007/s00432-022-04035-9 PMC11800847

[18] Rhodin KE, Raman V, Eckhoff A et al (2022) Patterns and Impact of fragmented care in stage II and III gastric cancer. Ann Surg Oncol 29(9):5422–5431. 10.1245/s10434-022-12031-z 35723791 10.1245/s10434-022-12031-z PMC9378672

[19] Hester CA, Karbhari N, Rich NE et al (2019) Effect of fragmentation of cancer care on treatment use and survival in hepatocellular carcinoma. Cancer 125(19):3428–3436. 10.1002/cncr.32336 31299089 10.1002/cncr.32336

[20] Freischlag K, Olivere L, Turner M, Adam M, Mantyh C, Migaly J (2021) Does fragmentation of care in locally advanced rectal cancer increase patient mortality? J Gastrointest Surg 25(5):1287–1296. 10.1007/s11605-020-04760-x 32754789 10.1007/s11605-020-04760-x

[21] Rhodin KE, Raman V, Jensen CW et al (2023) Multi-institutional care in clinical stage II and III esophageal cancer. Ann Thorac Surg 115(2):370–377. 10.1016/j.athoracsur.2022.06.049 35872035 10.1016/j.athoracsur.2022.06.049 PMC9851933

[22] Cham S, Huang Y, Melamed A et al (2021) Fragmentation of surgery and chemotherapy in the initial phase of ovarian cancer care and its association with overall survival. Gynecol Oncol 162(1):56–64. 10.1016/j.ygyno.2021.04.032 33965245 10.1016/j.ygyno.2021.04.032

[23] Khan H, Heslin MJ, Crook ED, Mehari K, Johnston FM, Fonseca AL (2022) Fragmentation of care in pancreatic cancer: effects on receipt of care and survival. J Gastrointest Surg 26(12):2522–2533. 10.1007/s11605-022-05478-8 36221020 10.1007/s11605-022-05478-8

[24] Mallin K, Browner A, Palis B et al (2019) Incident cases captured in the national cancer database compared with those in U.S. population based central cancer registries in 2012–2014. Ann Surg Oncol 26(6):1604–1612. 10.1245/s10434-019-07213-1 30737668 10.1245/s10434-019-07213-1

[25] American College of Surgeons. National cancer database. Updated 2020 https://www.facs.org/quality-programs/cancer-programs/national-cancer-database/ Accessed April 2 2024

[26] Korngold EK, Gollub MJ, Kim DH et al (2023) Update on the national accreditation program for rectal cancer (NAPRC): the radiologist’s role. Abdom Radiol 48(9):2814–2824. 10.1007/s00261-023-03919-9 10.1007/s00261-023-03919-9 37160474

[27] Chioreso C, Del Vecchio N, Schweizer ML, Schlichting J, Gribovskaja-Rupp I, Charlton ME (2018) Association between hospital and surgeon volume and rectal cancer surgery outcomes in patients with rectal cancer treated since 2000: systematic literature review and meta-analysis. Dis Colon Rectum 61(11):1320–1332. 10.1097/DCR.0000000000001198 30286023 10.1097/DCR.0000000000001198 PMC7000208

[28] Rhodin KE, Raman V, Jensen CW et al (2023) The effect of center esophagectomy volume on outcomes in clinical stage I to III esophageal cancer. Ann Surg 278(1):79–86. 10.1097/SLA.0000000000005681 36040026 10.1097/SLA.0000000000005681 PMC9971324

[29] Endo Y, Moazzam Z, Woldesenbet S et al (2023) Hospital volume and textbook outcomes in minimally invasive hepatectomy for hepatocellular carcinoma. J Gastrointest Surg 27(5):956–964. 10.1007/s11605-023-05609-9 36732402 10.1007/s11605-023-05609-9

[30] Doose M, Sanchez JI, Cantor JC et al (2021) Fragmentation of care among black women with breast cancer and comorbidities: the role of health systems. JCO Oncol Pract 17(5):e637–e644. 10.1200/OP.20.01089 33974834 10.1200/OP.20.01089 PMC8257967

[31] Vierra M, Bansal VV, Morgan RB et al (2024) Fragmentation of care in patients with peritoneal metastases undergoing cytoreductive surgery. Ann Surg Oncol 31(1):645–654. 10.1245/s10434-023-14318-1 37737968 10.1245/s10434-023-14318-1

[32] Bleicher RJ, Ruth K, Sigurdson ER et al (2016) Time to surgery and breast cancer survival in the United States. JAMA Oncol 2(3):330–339. 10.1001/jamaoncol.2015.4508 26659430 10.1001/jamaoncol.2015.4508 PMC4788555

[33] Brown ZJ, Labiner HE, Shen C, Ejaz A, Pawlik TM, Cloyd JM (2022) Impact of care fragmentation on the outcomes of patients receiving neoadjuvant and adjuvant therapy for pancreatic adenocarcinoma. J Surg Oncol 125(2):185–193. 10.1002/jso.26706 34599756 10.1002/jso.26706 PMC9113396

[34] Boffa DJ, Rosen JE, Mallin K et al (2017) Using the national cancer database for outcomes research: a review. JAMA Oncol 3(12):1722–1728. 10.1001/jamaoncol.2016.6905 28241198 10.1001/jamaoncol.2016.6905

[35] Lum SS, Browner AE, Palis B et al (2023) Disruption of national cancer database data models in the first year of the COVID-19 pandemic. JAMA Surg 158(6):643–650. 10.1001/jamasurg.2023.0652 37043215 10.1001/jamasurg.2023.0652

[36] Nogueira LM, Palis B, Boffa D, Lum S, Yabroff KR, Nelson H (2023) Evaluation of the impact of the COVID-19 pandemic on reliability of cancer surveillance data in the national cancer database. Ann Surg Oncol 30(4):2087–2093. 10.1245/s10434-022-12935-w 36539579 10.1245/s10434-022-12935-w PMC9767395

